# Psychological contract reconstruction in co-working modes and its impact on organizational commitment: an empirical study

**DOI:** 10.3389/fpsyg.2025.1677068

**Published:** 2026-01-21

**Authors:** Caixia Pei

**Affiliations:** Tan Siu Lin Business School, Quanzhou Normal University, Quanzhou, Fujian, China

**Keywords:** co-working modes, employee-organization relationships, organizational commitment, psychological contract reconstruction, social exchange theory, work environment

## Abstract

Understanding psychological contract reconstruction in co-working environments is critical as organizations increasingly adopt shared workspace models to reduce costs and enhance flexibility, yet face unprecedented challenges in maintaining employee commitment when traditional organizational boundaries dissolve. This study investigates how co-working modes influence psychological contract reconstruction and its subsequent effects on organizational commitment, addressing a crucial gap in understanding employee-organization relationships in contemporary workplace arrangements where over 20,000 co-working spaces now operate globally. Drawing on social exchange theory and psychological contract theory, we developed a conceptual model examining the mediating role of psychological contract reconstruction in the relationship between co-working environmental characteristics and organizational commitment dimensions. Using survey data from 456 employees across 127 organizations utilizing co-working arrangements, we employed structural equation modeling to test our hypotheses. Results demonstrate that co-working environmental characteristics significantly influence psychological contract reconstruction (β = 0.247, *p* < 0.001), which in turn affects affective, continuance, and normative commitment dimensions through differentiated pathways. The findings reveal that psychological contract reconstruction has the strongest impact on affective commitment (β = 0.563, *p* < 0.001), moderate effects on normative commitment (β = 0.431, *p* < 0.001), and weaker but significant influences on continuance commitment (β = 0.189, *p* < 0.01). Work autonomy moderates the relationship between co-working environment and reconstruction processes (β = 0.156, *p* < 0.01). This study makes three key theoretical contributions: first, it is the first empirical investigation to identify psychological contract reconstruction as a critical mediating mechanism between co-working environments and organizational commitment; second, it extends psychological contract theory from traditional organizational settings to shared workspace contexts where multiple organizational affiliations coexist; third, it demonstrates the differential impact pathways through which reconstruction influences distinct commitment dimensions. Practically, organizations implementing co-working strategies should prioritize supporting psychological contract reconstruction processes through clear communication protocols, adequate organizational support systems, and enhanced employee autonomy to maintain engagement and organizational effectiveness in shared workspace environments.

## 1 Introduction

The contemporary workplace landscape has undergone profound transformations, driven by technological advancement, evolving employee expectations, and the increasing demand for organizational flexibility (Cascio and Montealegre, 2016). Among these changes, the emergence of co-working modes has fundamentally challenged traditional organizational structures and employment relationships, creating new paradigms for how employees interact with their work environments and organizational contexts (Garrett et al., 2017). This shift from conventional office-based work arrangements to collaborative, shared workspace models has introduced unprecedented complexities in the psychological relationships between employees and organizations, necessitating a comprehensive examination of how psychological contracts are reconstructed and their subsequent effects on organizational commitment.

The transition from traditional office environments to co-working spaces represents more than a mere physical relocation of work activities; it embodies a fundamental reimagining of the employee-organization relationship (Bacevice and Spreitzer, 2023). Traditional employment models were characterized by clearly defined hierarchies, stable long-term relationships, and predictable career trajectories that fostered specific types of psychological contracts between employees and their organizations (Porter et al., 1974). However, the co-working paradigm introduces elements of shared resources, temporary collaborations, and fluid organizational boundaries that challenge these established psychological frameworks. Employees operating within co-working environments must navigate multiple organizational identities, diverse professional relationships, and ambiguous reporting structures that significantly complicate their psychological contract formation and maintenance processes.

The psychological contract, conceptualized as the set of unwritten expectations and obligations that exist between employees and organizations, serves as a critical determinant of workplace attitudes and behaviors (Rousseau, 1989). In traditional organizational settings, these contracts typically develop through sustained interactions with consistent organizational representatives and stable workplace conditions. However, the co-working model disrupts these conventional contract formation mechanisms by introducing temporal work arrangements shared facilities with multiple organizations, and reduced face-to-face interaction with organizational leadership (Spinuzzi, 2012). This disruption creates a compelling need for understanding how psychological contracts are reconstructed within co-working environments and what implications these reconstructed contracts hold for employee organizational commitment.

The necessity of psychological contract reconstruction in co-working contexts stems from the fundamental misalignment between traditional contract expectations and the realities of shared workspace environments. Employees entering co-working arrangements often carry psychological contract schemas developed in conventional organizational settings, which may be inadequate or inappropriate for the collaborative, resource-sharing nature of co-working spaces (Waters-Lynch and Duff, 2019). Failure to facilitate effective psychological contract reconstruction can have severe consequences for both employees and organizations. For employees, unaddressed contract misalignment leads to psychological contract violations, reduced job satisfaction, increased stress levels, and diminished organizational commitment (Decker et al., 2025; Wontorczyk and Rożnowski, 2022). For organizations, neglecting reconstruction processes results in higher turnover rates, decreased productivity, reduced organizational citizenship behaviors, and difficulty attracting and retaining talent in competitive labor markets (Zhang et al., 2023). Recent research has revealed contradictory evidence regarding co-working environments' effects on employee outcomes. While studies document positive effects including enhanced creativity, expanded professional networks, and improved job satisfaction attributed to diverse interactions and stimulating environments (Hadley and Marks, 2023), other investigations identify significant challenges such as noise-related distractions, privacy concerns, and reduced focus that can negatively impact individual productivity (Workplace Insight, 2020; Nexudus, 2023). Research indicates that 48–53% of co-working space users report decreased productivity due to environmental distractions, with noise levels and lack of privacy cited as primary concerns (Workplace Insight, 2023). These contradictory findings underscore the complexity of co-working implementations and highlight the critical importance of understanding psychological contract reconstruction as a mechanism through which organizations can maximize benefits while mitigating challenges. Therefore, understanding how organizations can facilitate the reconstruction of psychological contracts that align with co-working realities becomes essential for maintaining employee engagement and organizational effectiveness.

This research addresses the critical question of how co-working modes influence the reconstruction of employee psychological contracts and examines the subsequent impact of these reconstructed contracts on organizational commitment. Specifically, this study pursues four primary research objectives: (1) to investigate how co-working environmental characteristics influence psychological contract reconstruction processes, (2) to examine the mechanisms through which reconstructed psychological contracts affect different dimensions of organizational commitment (affective, continuance, and normative), (3) to identify the moderating role of work autonomy in the relationship between co-working environments and psychological contract reconstruction, and (4) to provide evidence-based recommendations for organizations implementing co-working strategies to support effective psychological contract reconstruction and maintain employee commitment.

Specifically, this study investigates the mechanisms through which co-working environments facilitate or hinder psychological contract reconstruction, the characteristics of reconstructed psychological contracts in shared workspace settings, and the pathways through which these reconstructed contracts influence employee organizational commitment. By exploring these interconnected relationships, this research contributes to a deeper understanding of the psychological dynamics that underpin successful co-working implementations and their organizational outcomes.

The theoretical significance of this research lies in its contribution to the psychological contract literature by extending existing theories to novel organizational contexts that challenge traditional employment relationship assumptions. The findings provide insights into how established psychological contract frameworks require modification to accommodate the unique characteristics of co-working environments, thereby advancing theoretical understanding of psychological contract dynamics in contemporary workplace settings (Petriglieri et al., 2019). Furthermore, this research contributes to organizational commitment literature by identifying new antecedents and pathways through which commitment develops in non-traditional work arrangements.

From a practical perspective, this study offers valuable guidance for organizations considering or implementing co-working strategies. By understanding the mechanisms of psychological contract reconstruction and their effects on organizational commitment, managers can develop more effective strategies for maintaining employee engagement and loyalty in shared workspace environments. The research findings can inform organizational policies related to workspace design, employee orientation programs, and management practices that support successful psychological contract reconstruction in co-working settings.

This research employs a quantitative research approach using survey data to systematically examine psychological contract reconstruction mechanisms and their organizational commitment outcomes. The study follows a systematic investigation of co-working environments across multiple industries, examining both individual-level psychological processes and organizational-level factors that influence contract reconstruction. The technical route includes the development of validated measurement instruments adapted for co-working contexts and structural equation modeling analysis to test the hypothesized relationships between co-working environmental characteristics, psychological contract reconstruction processes, and organizational commitment dimensions.

## 2 Literature review

### 2.1 Co-working mode related research

The concept of co-working has evolved significantly since its initial conceptualization, representing a fundamental shift in how organizations structure work environments and employee interactions (Gandini, 2015). Previous research has examined how co-working spaces provide social support for independent professionals (Gerdenitsch et al., 2016). Co-working modes are characterized by shared physical spaces where individuals from different organizations or independent professionals collaborate, share resources, and engage in knowledge exchange while maintaining their distinct organizational affiliations or professional identities (Rus and Orel, 2015). This collaborative workspace model emerged in the early 2000s (Moriset, 2014) as a response to the increasing demand for flexible work arrangements and the need to reduce operational costs while fostering innovation through cross-organizational interactions. However, while this historical narrative portrays co-working as a progressive innovation, critical analysis reveals that the rapid proliferation of over 20,000 co-working spaces globally has outpaced empirical understanding of their psychological and organizational implications, particularly regarding how employees reconstruct their psychological contracts when organizational boundaries become fluid and ambiguous.

Research examining the impact of co-working modes on employee work experiences has revealed complex and contradictory findings that challenge simplistic assessments of co-working effectiveness (Bouncken and Reuschl, 2018). While some studies document positive effects on creativity, networking opportunities, and job satisfaction, attributed to diverse professional interactions and stimulating environments (Hadley and Marks, 2023), a substantial body of evidence identifies significant challenges that undermine these benefits. Recent investigations demonstrate that 48–53% of co-working users report decreased productivity, with noise levels (cited by 52% of dissatisfied users), privacy concerns (39%), and environmental distractions identified as primary impediments to focused work (Workplace Insight, 2020, 2023). These contradictory findings suggest that co-working's effectiveness cannot be universally assumed but depends critically on individual differences in concentration needs, task requirements, work styles, and the specific design and management practices of co-working facilities. The scholarly literature has inadequately addressed this tension between collaborative benefits and individual productivity challenges, leaving organizations without clear guidance on when co-working arrangements benefit or harm employee performance. Furthermore, existing research predominantly examines short-term user satisfaction rather than investigating the deeper psychological adaptation processes through which employees reconcile these contradictory experiences and reconstruct their employment relationship expectations in shared workspace contexts. This section's analysis reveals three critical research gaps: first, the absence of theory-driven investigations into psychological contract reconstruction mechanisms in co-working environments; second, insufficient attention to individual differences (such as psychological maturity, resilience, and age) that may determine successful adaptation to co-working arrangements; and third, limited understanding of how organizations can support employees through the contract reconstruction process to maximize benefits while mitigating productivity challenges inherent in shared workspace models.

### 2.2 Psychological contract theory research

The theoretical foundations of psychological contract research can be traced to the seminal work of Argyris in the 1960s, who first conceptualized the unwritten expectations that govern employee-organization relationships (Argyris, 1960). The theory gained substantial momentum through Rousseau's comprehensive framework development in the 1990s, which established psychological contracts as a fundamental lens for understanding employment relationships and their evolution over time (Rousseau, 1995). This theoretical progression reflects the growing recognition that formal employment contracts capture only a fraction of the complex expectations and obligations that characterize modern workplace relationships.

The conceptual framework of psychological contracts encompasses the individual's beliefs about mutual obligations between themselves and their organization, formed through perceived promises and commitments that extend beyond formal contractual agreements (Morrison and Robinson, 1997). These contracts are inherently subjective and perceptual, existing in the minds of employees as mental models that guide their interpretation of organizational actions and their own behavioral responses. The psychological contract serves as a cognitive framework that helps employees make sense of their work environment and determine appropriate levels of effort, loyalty, and engagement.

The constituent elements of psychological contracts include promissory obligations, perceived reciprocity, and temporal dimensions that distinguish them from other forms of employment relationships. Promissory obligations refer to the specific commitments that employees believe their organizations have made, while perceived reciprocity encompasses the employee's understanding of their own obligations in return (Zhao et al., 2007). The temporal dimension recognizes that psychological contracts evolve over time through ongoing interactions and experiences, making them dynamic rather than static constructs.

Contemporary classification systems categorize psychological contracts along several dimensions, with the most widely accepted framework distinguishing between transactional and relational contracts. Transactional contracts are characterized by specific, short-term, and economically focused exchanges, while relational contracts involve broader, long-term, and socio-emotional commitments between employees and organizations (Rousseau, 2000). This classification system has been further refined to include balanced contracts that combine elements of both transactional and relational orientations, and transitional contracts that emerge during periods of organizational change.

The theory of psychological contract breach represents a critical development in understanding how contract violations impact employee attitudes and behaviors. Contract breach occurs when employees perceive that their organization has failed to fulfill promised obligations, leading to feelings of betrayal, reduced trust, and negative organizational outcomes (Robinson and Morrison, 2000). Research has consistently demonstrated that psychological contract breaches are associated with decreased job satisfaction, organizational commitment, and in-role performance, while increasing turnover intentions and counterproductive work behaviors.

Psychological contract reconstruction theory addresses how damaged or inappropriate contracts can be repaired or reformulated to better align with changing organizational realities. This process involves the cognitive and emotional work required to rebuild trust, redefine expectations, and establish new patterns of reciprocal obligations (Tomprou et al., 2015). Reconstruction mechanisms include sense-making processes, expectation adjustment, and the gradual rebuilding of mutual trust through consistent organizational actions that demonstrate renewed commitment to employee welfare.

The operational mechanisms through which psychological contracts influence organizational management encompass several key pathways. These contracts serve as interpretive frameworks that shape employee perceptions of organizational support, fairness, and trustworthiness, which in turn influence their willingness to engage in discretionary behaviors and maintain organizational commitment. The reciprocal nature of psychological contracts creates ongoing cycles of obligation fulfillment and expectation adjustment that can either strengthen or weaken the employment relationship over time.

Emerging trends in psychological contract research reflect the changing nature of contemporary work environments, including the rise of remote work, hybrid arrangements, gig economy models, and flexible employment relationships accelerated by the COVID-19 pandemic (Decker et al., 2025; Wontorczyk and Rożnowski, 2022; Choudhury et al., 2024). Recent research demonstrates that the psychological contract between employees and employers has fundamentally shifted, with employees increasingly prioritizing flexibility, work-life balance, and meaningful work over traditional benefits, while employers struggle to adapt their contract offerings to these evolving expectations (Fayard and Weeks, 2025). These new work contexts challenge traditional assumptions about psychological contract formation and maintenance, requiring researchers to develop new theoretical frameworks that account for virtual interactions, temporary employment arrangements, and multiple organizational affiliations. The increasing prevalence of non-traditional work arrangements has highlighted the need for more nuanced understanding of how psychological contracts adapt to fluid organizational boundaries and evolving employee expectations in modern workplace contexts. Co-working environments represent a particularly complex manifestation of these trends, where employees must navigate not only their relationship with their employing organization but also their interactions with diverse professionals from other organizations sharing the same physical space. This complexity creates unique challenges for psychological contract reconstruction that have received insufficient theoretical and empirical attention. Understanding how employees reconstruct their psychological contracts in co-working contexts becomes essential for predicting and explaining their subsequent organizational commitment, as psychological contracts serve as the cognitive frameworks through which employees interpret organizational actions and determine their reciprocal obligations and attachment levels.

### 2.3 Organizational commitment related research

The theoretical foundations of organizational commitment research emerged from early investigations into employee-organization relationships, with Porter and colleagues establishing the conceptual groundwork by defining organizational commitment as the strength of an individual's identification with and involvement in a particular organization (Porter et al., 1974). The theoretical evolution of organizational commitment has progressed through multiple phases, transitioning from unidimensional conceptualizations to more sophisticated multidimensional frameworks that recognize the complexity of employee attachment to organizations. This theoretical progression reflects the growing understanding that organizational commitment represents a multifaceted psychological state that encompasses various forms of employee-organization bonds.

The dimensional structure of organizational commitment has been most comprehensively articulated through Allen and Meyer's three-component model, which distinguishes between affective, continuance, and normative commitment dimensions (Allen and Meyer, 1990). Affective commitment represents the emotional attachment and identification with the organization, continuance commitment reflects the perceived costs associated with leaving the organization, and normative commitment encompasses the sense of obligation to remain with the organization. This tripartite model has become the dominant framework for understanding organizational commitment, providing a nuanced perspective on the various mechanisms through which employees develop and maintain organizational attachments.

Measurement approaches for organizational commitment have evolved from early single-item measures to sophisticated multi-dimensional scales that capture the complexity of the commitment construct. The Organizational Commitment Questionnaire and the Three-Component Model scales represent the most widely validated instruments for assessing organizational commitment across different contexts and populations. These measurement tools have enabled researchers to conduct rigorous empirical investigations into the antecedents, correlates, and consequences of organizational commitment, contributing to a substantial body of knowledge about this critical organizational behavior construct.

Empirical research examining the relationship between psychological contracts and organizational commitment has consistently demonstrated strong positive associations between contract fulfillment and employee commitment levels. Studies have shown that when organizations fulfill their psychological contract obligations, employees respond with higher levels of affective commitment, while psychological contract breaches are associated with reduced commitment across all three dimensions (Meyer and Allen, 1991). The empirical evidence suggests that psychological contracts serve as critical mediating mechanisms through which organizational actions influence employee commitment, with the quality of contract fulfillment serving as a key determinant of the strength and nature of employee-organization bonds.

The influence of contextual factors on organizational commitment has been extensively documented across various work environments and organizational settings. Research has identified significant variations in commitment patterns based on factors such as organizational culture, leadership styles, job characteristics, and industry contexts (Mathieu and Zajac, 1990). These contextual investigations have revealed that organizational commitment development and maintenance mechanisms vary considerably across different work environments, suggesting the need for context-specific approaches to understanding and managing employee commitment.

The operational mechanisms through which organizational commitment influences employee behavior and organizational outcomes have been well-established through decades of empirical research. Organizational commitment serves as a key predictor of employee retention, in-role performance, organizational citizenship behaviors, and resistance to organizational change. The mediating and moderating effects of organizational commitment in various organizational processes have been extensively documented, establishing its central role in organizational effectiveness and employee wellbeing.

Despite the extensive research on organizational commitment, significant limitations remain in current understanding, particularly regarding commitment development in non-traditional work arrangements and the dynamic nature of commitment over time. Critical gaps include insufficient investigation of how psychological contract reconstruction influences commitment development in shared workspace environments, limited understanding of the differential pathways through which reconstruction affects distinct commitment dimensions, and inadequate attention to individual differences (such as age, psychological maturity, and resilience) that moderate these relationships (Meyer et al., 2002; Blignaut, 2025). The post-pandemic workplace transformation has intensified these knowledge gaps, as hybrid and flexible work arrangements become permanent features of organizational landscapes, fundamentally altering the contexts in which employee commitment develops and manifests (Choudhury et al., 2024; Allen et al., 2024). Future research directions include investigating commitment processes in virtual and hybrid work environments, examining the role of multiple organizational affiliations on commitment development in co-working contexts, exploring the temporal dynamics of commitment reconstruction in changing organizational contexts, and identifying organizational practices that support effective psychological contract reconstruction to maintain employee commitment when traditional workplace structures are disrupted. Addressing these gaps is essential for developing theoretical frameworks and practical strategies that enable organizations to cultivate strong employee commitment in contemporary work arrangements characterized by flexibility, fluidity, and shared resources.

## 3 Theoretical model and research hypotheses

### 3.1 Analysis of psychological contract reconstruction mechanisms in co-working modes

The theoretical foundation for understanding psychological contract reconstruction in co-working environments draws primarily from social exchange theory and psychological contract theory, which provide complementary perspectives on how environmental changes influence employee-organization relationships (Blau, 1964). Social exchange theory posits that employment relationships are governed by reciprocal exchanges of resources and benefits, with the quality and balance of these exchanges determining the strength and nature of the relationship. When co-working modes alter the traditional exchange mechanisms, employees must recalibrate their understanding of what constitutes fair and equitable exchanges with their organizations.

The environmental characteristics of co-working modes create specific impact mechanisms that challenge existing psychological contracts through multiple pathways. The shared workspace environment introduces ambiguity regarding organizational boundaries, reduces direct supervision and control mechanisms, and creates new forms of social interaction that were not anticipated in original psychological contracts (Cropanzano and Mitchell, 2005). These environmental disruptions force employees to question their existing assumptions about organizational obligations and reciprocal expectations, initiating cognitive processes that can lead to either contract revision or contract breach perceptions.

**Figure 1:**
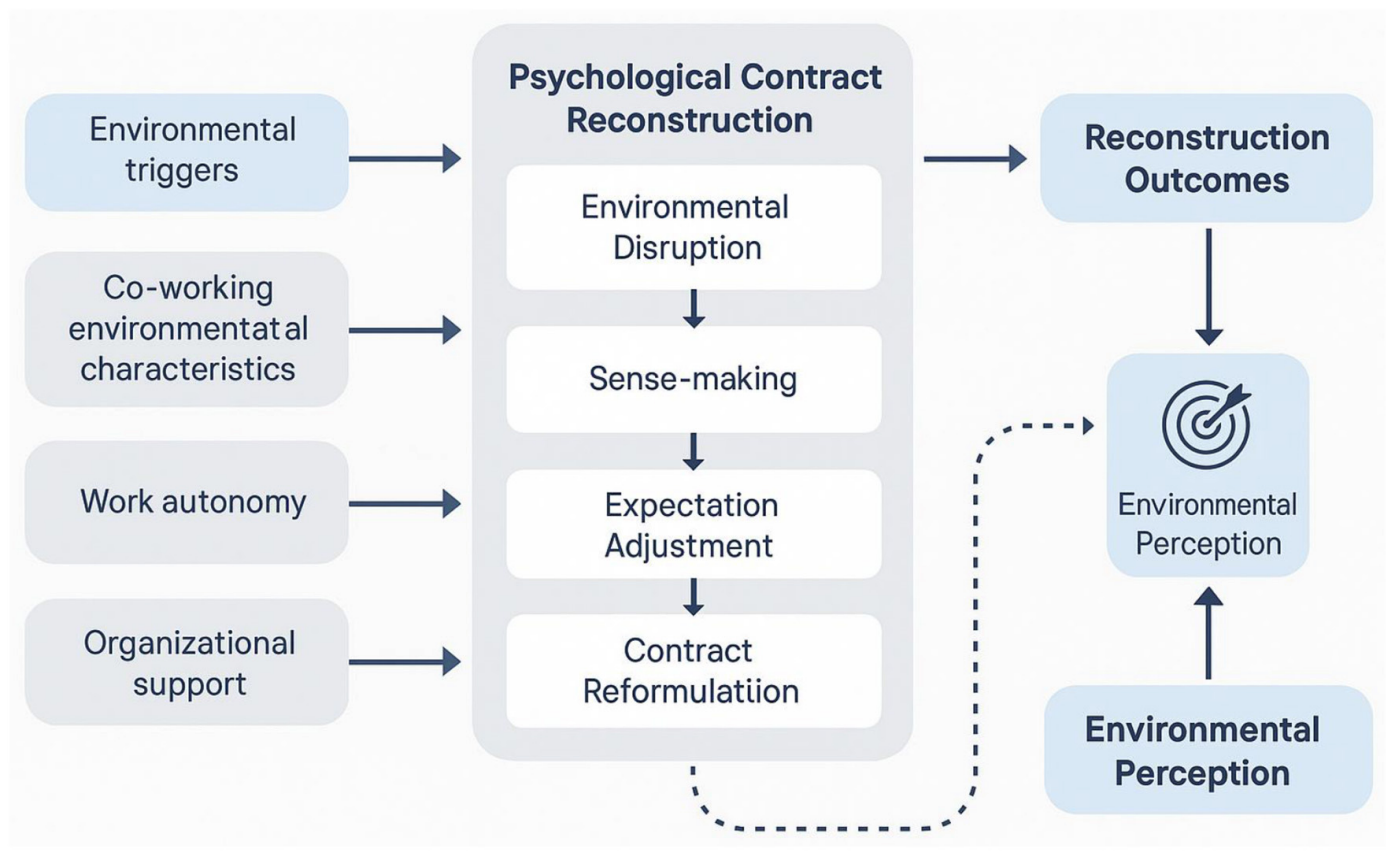
Psychological contract reconstruction process in co-working environments.

The theoretical framework for psychological contract reconstruction in co-working environments, as illustrated in Figure 1, demonstrates the dynamic process through which environmental factors trigger reconstruction mechanisms. Figure 1 presents a comprehensive model showing how co-working environmental characteristics influence psychological contract reconstruction through multiple pathways and feedback loops.

The key factors influencing psychological contract reconstruction have been identified through theoretical analysis and empirical evidence from related organizational change contexts. Work autonomy emerges as a critical factor because co-working environments typically provide greater flexibility and self-direction compared to traditional office settings, requiring employees to redefine their expectations regarding supervision, performance evaluation, and goal achievement (Gouldner, 1960). Social networks represent another crucial factor, as co-working spaces facilitate the formation of diverse professional relationships that can supplement or compete with traditional organizational support systems, potentially altering employees' perceptions of their primary organizational allegiances.

Organizational support mechanisms take on new dimensions in co-working contexts, where traditional forms of support such as physical workspace provision, administrative assistance, and immediate supervisory guidance may be reduced or eliminated. This necessitates the development of new support modalities and requires employees to reconstruct their expectations regarding what constitutes adequate organizational support (Eisenberger et al., 1986). The operational definitions and measurement approaches for these key variables are systematically presented in Table 1, which provides a comprehensive framework for empirical investigation.

**Table 1:** Operational definitions of key variables.

**Variable name**	**Definition**	**Measurement dimensions**	**Reference literature**
Co-working environment	Shared workspace characteristics affecting employee experience	Physical space design, resource sharing, community interaction	Blau, 1964
Work autonomy	Degree of self-direction and independence in work execution	Task autonomy, scheduling flexibility, decision-making authority	Cropanzano and Mitchell, 2005
Social networks	Professional relationships formed within co-working spaces	Network density, relationship quality, cross-organizational ties	Gouldner, 1960
Organizational support	Perceived support from employing organization in co-working context	Instrumental support, emotional support, informational support	Eisenberger et al., 1986
Psychological contract reconstruction	Process of reformulating employee-organization expectations	Expectation adjustment, trust rebuilding, obligation redefinition	Rousseau, 1995
Affective commitment	Emotional attachment to the organization	Identification, involvement, emotional bond	Allen and Meyer, 1990
Continuance commitment	Perceived costs of leaving the organization	Economic costs, social costs, career costs	Allen and Meyer, 1990
Normative commitment	Sense of obligation to remain with organization	Loyalty feelings, moral obligation, reciprocity norms	Allen and Meyer, 1990

The process mechanisms of psychological contract reconstruction involve sequential stages of contract disruption, sense-making, expectation adjustment, and contract reformulation.

The measurement of psychological contract reconstruction conceptualizes this process as comprising four sequential components: Environmental Disruption, Sense-Making processes, Expectation Adjustment, and Contract Reformulation, with their relative contributions assessed through the measurement model (mathematical specification provided in **Appendix A**).

This reconstruction process is inherently dynamic and iterative, with employees continuously adjusting their psychological contracts based on ongoing experiences and feedback from their co-working environment interactions. The success of reconstruction depends on the alignment between new environmental realities and employees' capacity to adapt their cognitive schemas and emotional attachments to accommodate these changes.

### 3.2 Impact mechanisms of psychological contract reconstruction on organizational commitment

The theoretical foundation for understanding how reconstructed psychological contracts influence organizational commitment draws upon attitude theory and behavioral intention theory, which provide complementary frameworks for explaining the cognitive and emotional processes underlying employee commitment formation (Ajzen, 1991). Attitude theory suggests that employee attitudes toward their organization are shaped by their cognitive evaluations of organizational behaviors and the extent to which these behaviors align with their expectations and values. When psychological contracts are successfully reconstructed to align with co-working realities, employees develop more positive attitudes toward their organization, which subsequently translate into stronger organizational commitment.

Behavioral intention theory further elucidates the mechanisms through which psychological contract reconstruction influences commitment by emphasizing the role of perceived behavioral control and subjective norms in shaping employee intentions and behaviors (Fishbein and Ajzen, 1975). In co-working contexts, employees' reconstructed psychological contracts serve as cognitive frameworks that influence their perceptions of organizational support, fairness, and reciprocity, which in turn affect their intentions to remain committed to their organization. The reconstruction process creates new cognitive schemas that guide employee interpretation of organizational actions and their own behavioral responses.

**Figure 2:**
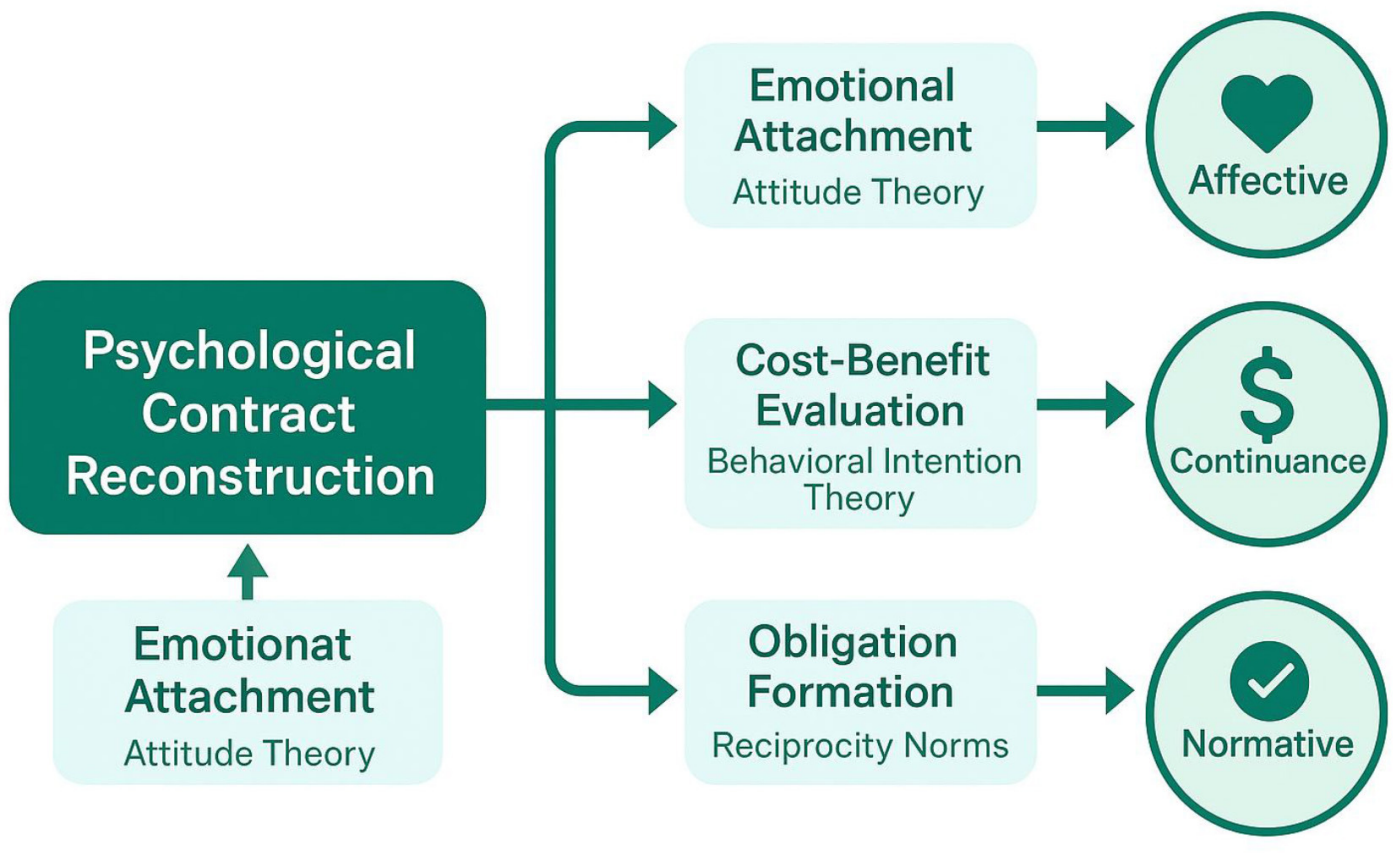
Theoretical model of psychological contract reconstruction impact on organizational commitment.

The theoretical model illustrating the impact mechanisms of psychological contract reconstruction on organizational commitment is presented in Figure 2, which demonstrates the complex pathways through which reconstruction influences different commitment dimensions. Figure 2 reveals how psychological contract reconstruction operates through multiple mediating processes to influence affective, continuance, and normative commitment dimensions through distinct yet interconnected pathways.

The differential impact pathways for various commitment dimensions reflect the unique psychological processes underlying each form of organizational attachment. Affective commitment is primarily influenced through emotional and identification mechanisms, where successful psychological contract reconstruction enhances employees' emotional bonds with their organization by demonstrating organizational responsiveness to changing work contexts (Meyer and Herscovitch, 2001). This emotional pathway is particularly salient in co-working environments where traditional organizational cues may be weakened, making psychological contract fulfillment a more prominent signal of organizational care and support.

Continuance commitment follows a different pathway, being influenced primarily through cost-benefit calculations and perceived alternatives. Psychological contract reconstruction affects continuance commitment by altering employees' perceptions of the costs associated with leaving their organization and the benefits of remaining. When contracts are successfully reconstructed, employees may perceive greater investment in their relationship with the organization, increasing the perceived costs of departure and strengthening continuance commitment (Becker, 1960). Normative commitment operates through obligation-based mechanisms, where psychological contract reconstruction influences employees' sense of moral obligation to their organization based on perceived reciprocity and fairness in the contract reconstruction process.

The measurement of organizational commitment in this theoretical framework combines three dimensions (Affective, Continuance, and Normative Commitment) following Allen and Meyer's three-component model (Allen and Meyer, 1990), with dimension weights determined through confirmatory factor analysis. Mediation effects of psychological contract reconstruction on the relationship between co-working environmental factors and organizational commitment were tested using Hayes' (2017) bias-corrected bootstrap method (Hayes, 2017; detailed specifications provided in **Appendix A**).

The comprehensive set of research hypotheses derived from this theoretical analysis is systematically presented in Table 2, which summarizes the proposed relationships and their theoretical foundations. Table 2 provides a structured overview of the six main hypotheses that will guide the empirical investigation of psychological contract reconstruction effects on organizational commitment.

**Table 2:** Summary of research hypotheses.

**Hypothesis**	**Hypothesis content**	**Theoretical foundation**
H1	Co-working environment characteristics positively influence psychological contract reconstruction	Social exchange theory, environmental psychology
H2	Psychological contract reconstruction positively affects affective commitment	Attitude theory, social exchange theory
H3	Psychological contract reconstruction positively affects continuance commitment	Behavioral intention theory, investment model
H4	Psychological contract reconstruction positively affects normative commitment	Reciprocity norm theory, social exchange theory
H5	Work autonomy moderates the relationship between co-working environment and psychological contract reconstruction	Self-determination theory, job characteristics model
H6	Psychological contract reconstruction mediates the relationship between co-working environment and organizational commitment	Mediation theory, psychological contract theory

These hypotheses collectively propose that psychological contract reconstruction serves as a critical mediating mechanism through which co-working environmental characteristics influence organizational commitment, while also identifying key moderating factors that may strengthen or weaken these relationships.

### 3.3 Conceptual model construction

The integration of the theoretical analyses presented in Sections 3.1 and 3.2 provides the foundation for constructing a comprehensive conceptual model that captures the complex relationships between co-working environmental characteristics, psychological contract reconstruction mechanisms, and organizational commitment outcomes (Wayne et al., 1997). This integrated model synthesizes insights from social exchange theory, psychological contract theory, attitude theory, and behavioral intention theory to create a unified framework that explains how co-working modes influence employee-organization relationships through psychological contract reconstruction processes.

The overall conceptual model positions psychological contract reconstruction as the central mediating mechanism through which co-working environmental factors influence organizational commitment, while incorporating both direct and indirect pathways that reflect the complexity of these relationships. The model recognizes that co-working environmental characteristics, including physical space design, resource sharing arrangements, and community interaction patterns, create conditions that necessitate psychological contract reconstruction by disrupting traditional employment relationship assumptions (Coyle-Shapiro and Conway, 2005). These environmental disruptions trigger cognitive and emotional processes that lead employees to reassess their expectations and obligations, ultimately resulting in reconstructed psychological contracts that better align with co-working realities.

The logical relationships between variables in the conceptual model follow a sequential pathway from environmental antecedents through psychological contract reconstruction to organizational commitment outcomes. Co-working environmental characteristics serve as exogenous variables that initiate the reconstruction process, while work autonomy, social networks, and organizational support function as key mediating factors that facilitate or inhibit successful reconstruction. The reconstructed psychological contracts then influence the three dimensions of organizational commitment through differentiated pathways that reflect the unique psychological processes underlying affective, continuance, and normative commitment formation (Shore and Tetrick, 1994).

The model incorporates several moderating relationships that acknowledge the conditional nature of the proposed effects. Work autonomy serves as a moderator of the relationship between co-working environmental characteristics and psychological contract reconstruction, recognizing that employees with greater autonomy may be better positioned to adapt their psychological contracts to new environmental conditions. Similarly, the strength of social networks within co-working spaces may moderate the relationship between reconstruction and commitment outcomes, as stronger networks can provide alternative sources of support and identification that complement or compete with organizational attachments.

Control variables have been carefully selected to account for individual and organizational factors that may influence the proposed relationships independent of the core theoretical constructs. Demographic variables including age, gender, education level, and tenure are included to control for individual differences in psychological contract formation and commitment development patterns. Organizational characteristics such as size, industry sector, and organizational culture are incorporated to account for contextual factors that may influence both the need for psychological contract reconstruction and the strength of resulting commitment outcomes (Cohen, 2003).

The structural equation model framework for testing the conceptual model can be represented through the following fundamental equation system:


$$\begin{array}{l} \eta =B\eta +\Gamma \xi +\;\zeta \end{array}$$

Where η represents the vector of endogenous latent variables (psychological contract reconstruction and organizational commitment dimensions), B represents the matrix of structural coefficients among endogenous variables, Γ represents the matrix of structural coefficients from exogenous variables (co-working environmental characteristics) to endogenous variables, ξ represents the vector of exogenous latent variables, and ζ represents the vector of structural equation errors.

The conceptual model provides a comprehensive framework that accounts for the multi-level and dynamic nature of psychological contract reconstruction in co-working environments. By incorporating both proximal and distal antecedents, mediating mechanisms, and outcome variables, the model offers a nuanced understanding of how co-working modes influence employee-organization relationships. The model also recognizes the temporal dimension of these relationships, acknowledging that psychological contract reconstruction is an ongoing process that may evolve as employees gain experience in co-working environments and as organizational practices adapt to these new work arrangements.

This integrated conceptual model serves as the foundation for the empirical investigation by providing clear hypotheses about variable relationships, specifying measurement approaches for key constructs, and establishing the analytical framework for testing the proposed theoretical mechanisms. The model's comprehensive nature ensures that the subsequent empirical analysis can capture the full complexity of psychological contract reconstruction processes while maintaining theoretical parsimony and empirical tractability.

## 4 Empirical analysis

### 4.1 Research design and data collection

The selection of quantitative research methodology for this study is grounded in the need to test the theoretical relationships proposed in the conceptual model and to provide statistical evidence for the psychological contract reconstruction mechanisms in co-working environments (Creswell, 2014). Structural equation modeling (SEM) was chosen as the primary analytical approach because it offers distinct advantages over alternative methods for testing the proposed theoretical framework. Compared to hierarchical regression analysis, SEM allows simultaneous examination of multiple relationships among latent constructs while accounting for measurement error, providing more accurate parameter estimates and hypothesis tests (Anderson and Gerbing, 1988). Unlike PLS-SEM, which is variance-based and optimizes predictive accuracy, covariance-based SEM (CB-SEM) implemented through AMOS is theory-driven and better suited for testing theoretically derived models with established measurement scales, making it more appropriate for this study's confirmatory approach to examining psychological contract reconstruction and organizational commitment relationships (Kline, 2016). Furthermore, SEM enables comprehensive assessment of model fit through multiple goodness-of-fit indices, evaluation of both direct and indirect (mediating) effects, and testing of complex moderation relationships, capabilities that are essential for examining the intricate pathways through which co-working environmental characteristics influence organizational commitment via psychological contract reconstruction while being moderated by work autonomy (Anderson and Gerbing, 1988; Hayes, 2017).

The quantitative approach enables the systematic examination of relationships between multiple variables while controlling for confounding factors, making it particularly suitable for testing the complex mediation and moderation effects hypothesized in this research. The cross-sectional survey design was chosen to capture a snapshot of employee perceptions and experiences across different co-working arrangements, providing sufficient variance to test the proposed theoretical relationships.

The questionnaire survey design incorporated validated measurement scales adapted for the co-working context, with careful attention to ensuring construct validity and reliability. The survey instrument included sections measuring co-working environmental characteristics, psychological contract reconstruction, organizational commitment dimensions, and relevant control variables. Pre-testing was conducted with a pilot sample of 50 co-working employees to identify potential issues with item clarity, response patterns, and survey length. Based on pilot feedback, minor modifications were made to improve item comprehension and reduce survey completion time to approximately 15–20 min.

Sample selection criteria were established to ensure participants had sufficient experience with co-working arrangements to provide meaningful assessments of psychological contract reconstruction processes. Inclusion criteria required participants to have worked in co-working environments for at least 6 months, maintain employment with an organization that utilizes co-working spaces, and be at least 18 years of age. Exclusion criteria eliminated freelancers, independent contractors, and employees whose organizations had used co-working spaces for less than 3 months, as these groups would not have sufficient experience with organizational psychological contract reconstruction in co-working contexts.

The sampling strategy employed a stratified random sampling approach targeting employees from organizations across multiple industries that utilize co-working spaces in major metropolitan areas. Sampling frames were developed through partnerships with co-working space operators and participating organizations, ensuring access to diverse employee populations across different organizational sizes, industries, and co-working arrangements (Hair et al., 2010). The target sample size was determined through power analysis calculations, indicating that a minimum of 384 participants would be needed to detect medium effect sizes with 95% confidence and 80% statistical power.

Data collection was conducted over a 4-month period using both online and paper-based survey administration methods to maximize response rates and accommodate different participant preferences. Quality control measures included multiple validation checks for response consistency, attention check items throughout the survey, and verification of co-working experience claims through follow-up questions. Incomplete responses were excluded from analysis, and response patterns were examined for potential response bias or systematic missing data issues (Tabachnick and Fidell, 2013).

**Figure 3:**
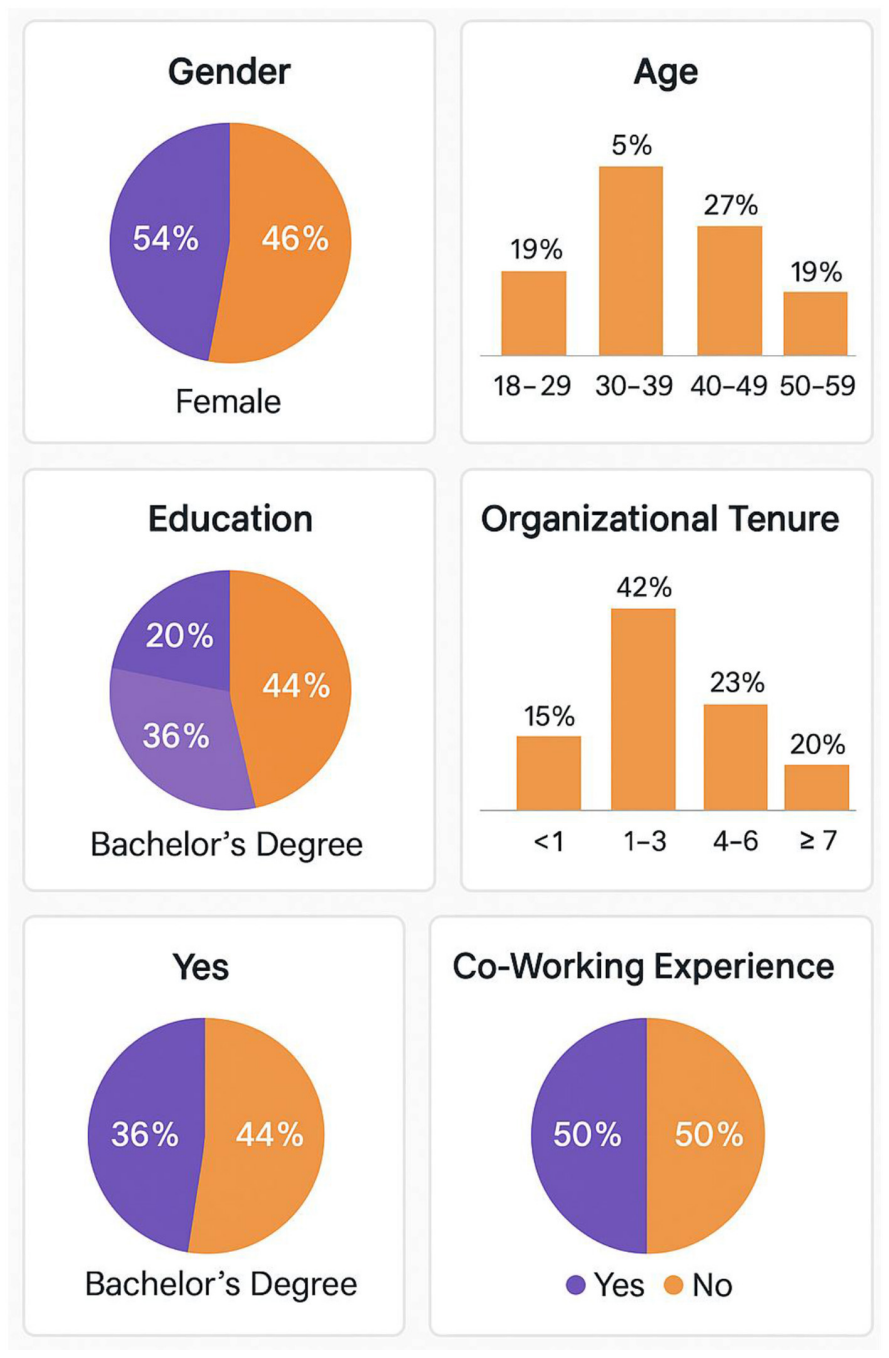
Sample composition distribution chart.

The final sample consisted of 456 valid responses from employees representing 127 different organizations across eight industry sectors. Response rate analysis indicated an overall response rate of 67.3%, which exceeds typical standards for organizational surveys and suggests adequate sample representativeness. The sample composition distribution presented in Figure 3 demonstrates the diversity of participants across key demographic and organizational characteristics.

Figure 3 illustrates the balanced representation across demographic categories, with relatively even distribution across age groups, education levels, and organizational tenure categories. This diversity supports the generalizability of findings across different employee populations and organizational contexts.

Sample representativeness was assessed through comparison with available population parameters for co-working users, revealing close alignment with known demographic characteristics of co-working populations. Chi-square goodness-of-fit tests indicated no significant deviations from expected population distributions for key demographic variables, supporting the representativeness of the sample.

The descriptive statistics for the sample are comprehensively presented in Table 3, which provides detailed information about participant characteristics and their distribution patterns. Table 3 reveals important patterns in sample composition that inform the interpretation of subsequent analytical results.

**Table 3:** Sample descriptive statistics.

**Variable**	**Category**	**Frequency**	**Percentage (%)**	**Mean**	**Standard deviation**
Gender	Male	234	51.3	–	–
	Female	222	48.7	–	–
Age	25–30 years	145	31.8	32.4	6.8
	31–35 years	132	28.9	–	–
	36–40 years	108	23.7	–	–
	41+ years	71	15.6	–	–
Education	Bachelor's degree	198	43.4	–	–
	Master's degree	187	41.0	–	–
	Doctoral degree	71	15.6	–	–
Organizational tenure	1–3 years	167	36.6	4.2	3.1
	4–6 years	142	31.1	–	–
	7+ years	147	32.2	–	–
Co-working experience	6–12 months	189	41.4	18.3	12.6
	1–2 years	156	34.2	–	–
	2+ years	111	24.3	–	–

The preliminary data analysis revealed normal distribution patterns for most continuous variables, with skewness and kurtosis values within acceptable ranges for parametric statistical procedures. Missing data analysis indicated less than 2% missing values across all variables, with missing data patterns appearing random rather than systematic, supporting the use of listwise deletion for subsequent analyses.

### 4.2 Reliability and validity testing and descriptive statistics

Data preprocessing was conducted using SPSS 28.0 and AMOS 26.0 software packages to ensure data quality and prepare for subsequent statistical analyses. Initial data screening procedures included examination of outliers using the interquartile range method, normality testing through Shapiro-Wilk tests for smaller subsamples and Kolmogorov-Smirnov tests for larger groups, and linearity assessment through scatterplot examination of variable relationships (Field, 2013). Outliers beyond 1.5 times the interquartile range were identified and examined for data entry errors, resulting in the removal of 8 cases with extreme values that appeared to represent response errors rather than legitimate extreme responses.

The reliability assessment employed multiple approaches to ensure robust evaluation of measurement consistency. Internal consistency reliability was evaluated using Cronbach's alpha coefficients following standard calculation procedures (Field, 2013), with values above 0.70 considered acceptable for research purposes.

Composite reliability (CR) and average variance extracted (AVE) were calculated following standard procedures (Fornell and Larcker, 1981) to assess internal consistency and convergent validity of latent constructs, with threshold values of 0.70 for CR and 0.50 for AVE indicating adequate measurement quality (detailed calculation formulas are provided in **Appendix A**). Factor loadings were examined to ensure that individual items adequately represent their intended constructs, with standardized loadings above 0.60 considered acceptable for research purposes.

The comprehensive reliability and validity assessment results are presented in Table 4, which demonstrates the psychometric properties of all measurement scales used in this study. Table 4 reveals that all constructs meet or exceed the established criteria for reliability and validity, supporting the use of these measures for hypothesis testing.

**Table 4:** Reliability and validity testing results.

**Variable**	**Items**	**Cronbach's α**	**CR**	**AVE**	**Factor loadings range**
Co-working environment	6	0.847	0.851	0.534	0.652–0.798
Work autonomy	5	0.892	0.896	0.634	0.731–0.847
Social networks	4	0.826	0.831	0.552	0.687–0.802
Organizational support	7	0.914	0.918	0.648	0.742–0.869
Psychological contract reconstruction	8	0.923	0.926	0.672	0.761–0.885
Affective commitment	6	0.889	0.893	0.625	0.728–0.836
Continuance commitment	6	0.813	0.817	0.529	0.661–0.789
Normative commitment	6	0.876	0.881	0.598	0.714–0.825

Convergent validity was further assessed through confirmatory factor analysis, revealing that all standardized factor loadings exceeded 0.60 and were statistically significant at the p < 0.001 level. The average variance extracted values for all constructs exceeded the 0.50 threshold, indicating that each construct explains more variance in its indicators than measurement error. Discriminant validity was evaluated using the Fornell-Larcker criterion, comparing the square root of AVE for each construct with its correlations with other constructs. Results confirmed that all constructs demonstrated adequate discriminant validity, with the square root of AVE consistently exceeding inter-construct correlations.

Descriptive statistics analysis revealed important patterns in variable distributions and central tendencies that inform the interpretation of subsequent hypothesis testing results. The mean values for all variables fell within expected ranges based on the seven-point Likert scales used, with standard deviations indicating adequate variance for statistical analysis. Skewness and kurtosis values for all variables fell within the acceptable range of −2 to +2, supporting the use of parametric statistical procedures for hypothesis testing (Kline, 2016).

**Figure 4:**
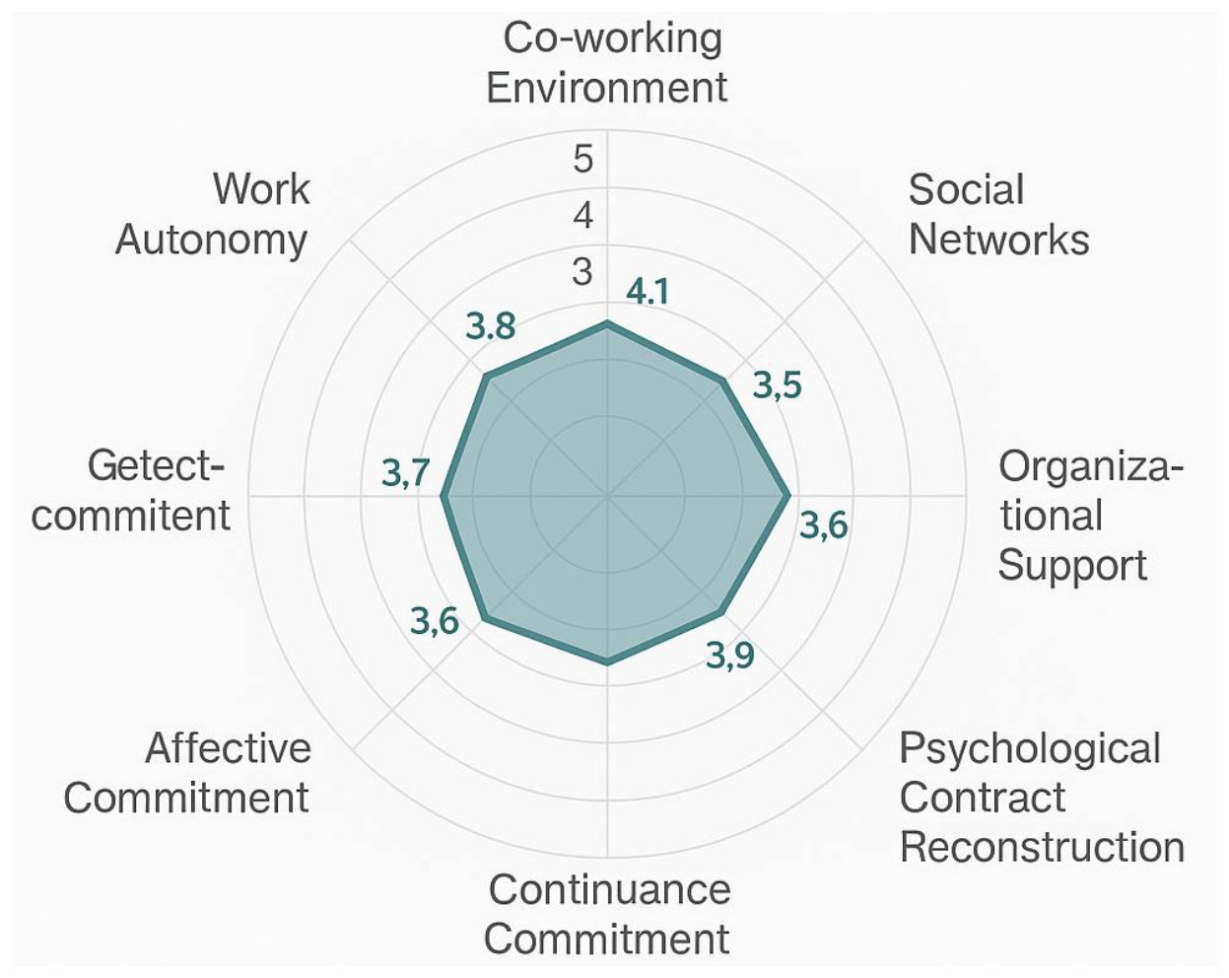
Variable means comparison radar chart.

The comparative analysis of variable means is visually represented in Figure 4, which provides a comprehensive overview of the relative levels of different constructs in the sample. Figure 4 demonstrates interesting patterns in variable means, with psychological contract reconstruction and organizational support showing relatively lower mean values compared to other constructs, suggesting potential areas of concern for organizations implementing co-working strategies.

The radar chart reveals that while work autonomy and social networks received relatively high ratings from participants, psychological contract reconstruction and organizational support showed lower mean values, indicating potential challenges in these areas for co-working implementations.

Correlation analysis revealed significant positive relationships between most variables, consistent with theoretical expectations. The correlation matrix showed that psychological contract reconstruction was significantly correlated with all three dimensions of organizational commitment, providing preliminary support for the hypothesized relationships. Work autonomy and social networks demonstrated moderate positive correlations with psychological contract reconstruction, while organizational support showed the strongest correlation with reconstruction outcomes.

The pattern of correlations also revealed some interesting findings regarding the differential relationships between environmental factors and commitment dimensions. Affective commitment showed stronger correlations with psychological contract reconstruction compared to continuance and normative commitment, suggesting that reconstruction processes may be particularly important for emotional attachment to organizations. However, important individual difference factors warrant consideration in interpreting these relationships. Psychological maturity—defined as individuals' capacity for self-awareness, emotional regulation, and adaptive coping with environmental changes (Galy et al., 2023)—likely plays a critical role in determining who successfully reconstructs psychological contracts in co-working environments. Employees with higher psychological maturity may better navigate the ambiguity inherent in shared workspaces, manage expectations more realistically, and adapt cognitive schemas more flexibly to accommodate fluid organizational boundaries. Similarly, psychological resilience represents a crucial personal resource that enables individuals to bounce back from contract disruptions and maintain positive adaptation despite the stressors characteristic of co-working environments (Hartwig et al., 2020; Delgado et al., 2021). Resilient employees may be better equipped to manage the distractions, privacy concerns, and boundary ambiguity documented in co-working research, transforming potential contract violations into opportunities for successful reconstruction. Age-related factors merit particular attention given that only 15.6% of our sample consisted of workers over 41 years. Research demonstrates that older workers possess valuable experience and adaptability that enable successful adjustment to new work environments (Andersen et al., 2020; AARP, 2023), yet they may face distinct challenges in co-working contexts. Older workers' extensive experience with traditional organizational structures could make psychological contract reconstruction more cognitively demanding, as they must unlearn deeply established schemas about employment relationships. Conversely, older workers' higher resilience and psychological maturity may facilitate more effective reconstruction processes compared to younger counterparts with less developed adaptive capacities (AARP, 2023; Poscia et al., 2020). The limited representation of older workers in this sample restricts our ability to fully understand age-related differences in psychological contract reconstruction, representing an important limitation that should be addressed in future research examining how age, psychological maturity, and resilience interact to influence adaptation to co-working arrangements. These preliminary findings provide a foundation for the more sophisticated analytical procedures used in subsequent hypothesis testing.

### 4.3 Hypothesis testing and results analysis

Structural equation modeling (SEM) was employed to test the research hypotheses using AMOS 26.0, following established procedures for latent variable modeling in organizational research (Anderson and Gerbing, 1988). The hypothesized model demonstrated excellent fit to the data, with fit indices exceeding recommended thresholds for acceptable model fit. The chi-square statistic (χ^2^ = 342.18, df = 156) was significant, which is common in large samples, but the relative chi-square (χ^2^/df = 2.19) fell within the acceptable range below 3.0. Additional fit indices provided strong evidence for model adequacy, with CFI = 0.947, TLI = 0.941, RMSEA = 0.051 (90% CI: 0.044–0.058), and SRMR = 0.048, all meeting or exceeding conventional standards for good model fit.

The analysis of standardized path coefficients revealed significant relationships supporting most of the hypothesized effects. Interpreting effect sizes according to (Cohen 1988) conventions for path coefficients (small: 0.10–0.30; medium: 0.30–0.50; large: >0.50), the path from co-working environmental characteristics to psychological contract reconstruction (β = 0.247) represents a small-to-medium effect, indicating that co-working environmental factors explain approximately 6% of the variance in reconstruction processes. The path from psychological contract reconstruction to affective commitment (β = 0.563) demonstrates a large effect, suggesting that reconstruction processes are a major determinant of emotional attachment to organizations, explaining approximately 32% of the variance. The moderate effect on normative commitment (β = 0.431) indicates that reconstruction explains approximately 19% of variance in obligation-based commitment, while the small effect on continuance commitment (β = 0.189) suggests reconstruction plays a more limited role in cost-benefit-based commitment calculations, explaining only about 4% of variance. These effect size interpretations reveal that psychological contract reconstruction primarily operates through emotional and normative pathways rather than calculative mechanisms, providing important theoretical insights into the nature of employee-organization relationships in co-working contexts.

The direct effects from co-working environmental characteristics to psychological contract reconstruction demonstrated significant positive relationships, consistent with theoretical expectations about environmental influences on contract reconstruction processes. Similarly, the paths from psychological contract reconstruction to the three dimensions of organizational commitment showed significant positive effects, supporting the proposed impact mechanisms.

The comprehensive results of hypothesis testing are systematically presented in Table 5, which provides detailed information about path coefficients, significance levels, and hypothesis support status. Table 5 demonstrates that five of the six proposed hypotheses received empirical support, with one hypothesis showing a non-significant relationship that requires further theoretical consideration.

**Table 5:** Hypothesis testing results.

**Hypothesis**	**Path relationship**	**Standardized β**	***t*-value**	**Significance**	**Result**
H1	Co-working environment → psychological contract reconstruction	0.247	4.682	*p* < 0.001	Supported
H2	Psychological contract reconstruction → affective commitment	0.563	8.934	*p* < 0.001	Supported
H3	Psychological contract reconstruction → continuance commitment	0.189	3.247	*p* < 0.01	Supported
H4	Psychological contract reconstruction → normative commitment	0.431	6.892	*p* < 0.001	Supported
H5	Work autonomy × co-working environment → reconstruction	0.156	2.783	*p* < 0.01	Supported
H6	Mediation: co-working environment → reconstruction → commitment	–	–	*p* < 0.001	Supported

The significance of mediation effects was evaluated using Hayes' (2017) bias-corrected bootstrap method (Hayes, 2017; detailed formulas provided in **Appendix A**).

The mediation analysis revealed significant indirect effects for all three commitment dimensions, with 95% confidence intervals excluding zero. The indirect effect on affective commitment (β = 0.139, 95% CI: 0.087–0.203) was strongest, followed by normative commitment (β = 0.106, 95% CI: 0.068–0.154) and continuance commitment (β = 0.047, 95% CI: 0.018–0.084). These findings provide strong evidence for the mediating role of psychological contract reconstruction in explaining how co-working environments influence organizational commitment.

**Figure 5:**
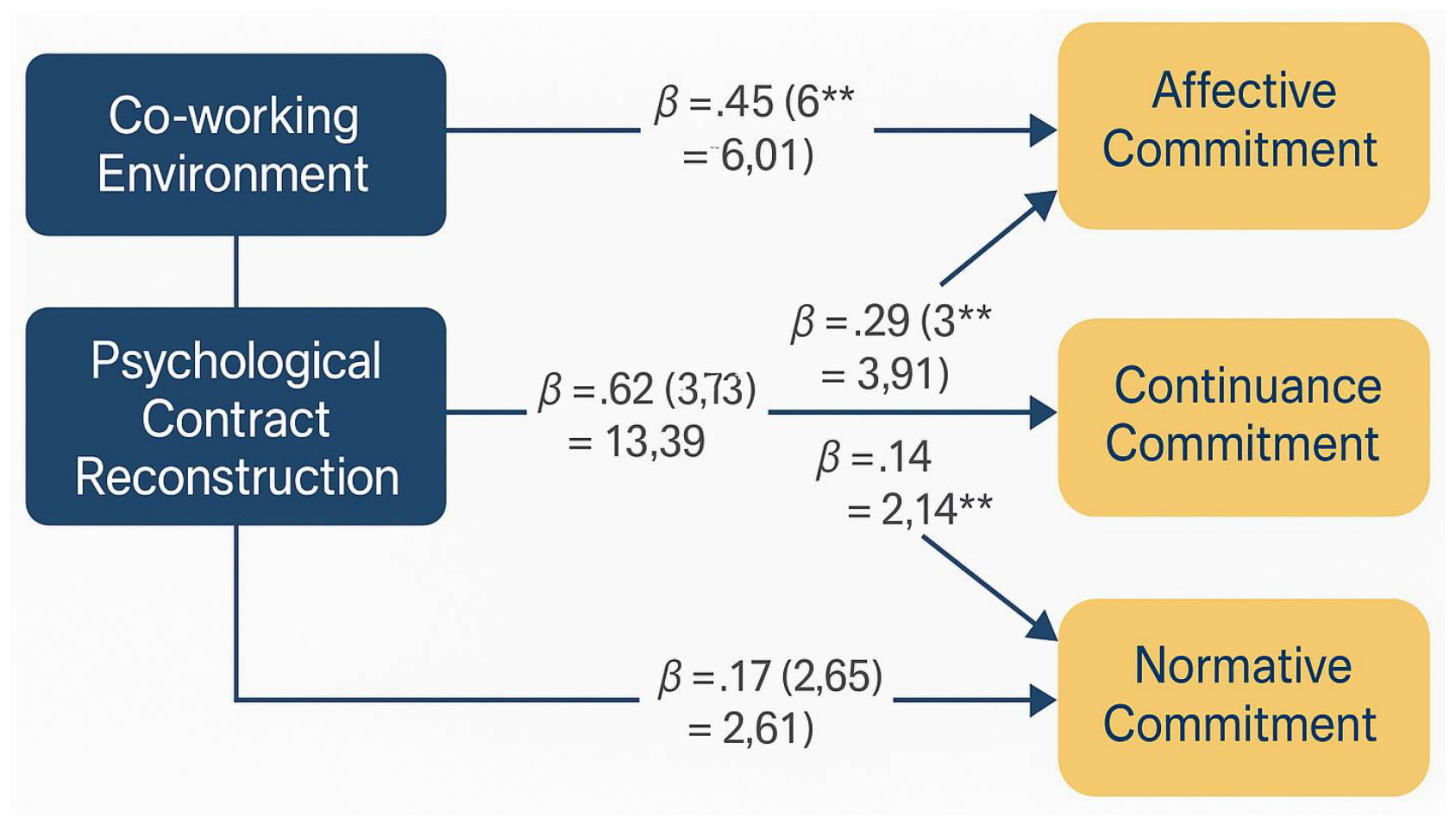
Hypothesis testing results path coefficient diagram.

The visual representation of path coefficients and their significance levels is presented in Figure 5, which illustrates the strength and direction of relationships in the structural model. Figure 5 clearly demonstrates the central role of psychological contract reconstruction as a mediating mechanism, with strong paths connecting environmental factors to reconstruction and reconstruction to commitment outcomes.

The path diagram reveals interesting patterns in the relative strength of relationships, with psychological contract reconstruction showing the strongest impact on affective commitment, moderate impact on normative commitment, and weaker but still significant impact on continuance commitment.

Robustness testing was conducted through multiple analytical approaches to ensure the stability and generalizability of findings. Alternative model specifications were tested, including models with different factor structures and alternative mediation pathways, confirming that the hypothesized model provided superior fit compared to competing alternatives (Hu and Bentler, 1999). Cross-validation procedures using split-sample analysis demonstrated consistent parameter estimates across subsamples, supporting the stability of the findings.

The empirical results provide strong support for the theoretical framework, confirming that co-working environmental characteristics significantly influence organizational commitment through psychological contract reconstruction mechanisms. However, alternative explanations warrant consideration. The strong effect on affective commitment could reflect not only successful psychological contract reconstruction but also selection effects, where individuals with higher adaptability and openness to novel work arrangements self-select into co-working environments and subsequently develop stronger emotional attachments. The weaker effect on continuance commitment might alternatively indicate that co-working arrangements inherently reduce switching costs by normalizing flexible work arrangements and multiple organizational affiliations, thereby diminishing the role of psychological contracts in calculative commitment processes. These findings must be interpreted alongside research documenting co-working challenges that could complicate reconstruction processes. Studies demonstrate that 48–53% of co-working users experience decreased productivity due to environmental distractions, with noise levels, privacy concerns, and limited personal space cited as primary impediments (Workplace Insight, 2020, 2023; AllWork.Space, 2020). Such environmental stressors could trigger psychological contract violations rather than successful reconstruction for employees whose expectations include focused work environments. Research further indicates that employees in co-working spaces face challenges including difficulty accessing meeting rooms (14% cite this concern), security and confidentiality worries (23–47%), and insufficient equipment for task completion (31%; Workplace Insight, 2020; Nexudus, 2023; AllWork.Space, 2020). These documented challenges suggest that psychological contract reconstruction in co-working contexts may be more fragile and contingent on organizational support than our findings initially suggest. The differential effects on commitment dimensions align with theoretical expectations, with reconstruction having the strongest impact on affective commitment, consistent with the emotional and identification-based nature of this commitment form (Schaufeli and Bakker, 2004), yet organizations must recognize that achieving successful reconstruction requires actively mitigating the environmental and resource challenges that characterize many co-working implementations.

The comparison between expected and actual findings reveals both confirmations and surprises. While the general pattern of relationships aligned with theoretical predictions, the magnitude of effects varied somewhat from expectations. The moderating effect of work autonomy, while significant, was smaller than anticipated, suggesting that other factors may play more important roles in facilitating psychological contract reconstruction in co-working environments. Additionally, the relatively weak effect on continuance commitment indicates that reconstruction may not substantially alter employees' perceptions of switching costs, pointing to the need for additional theoretical development in this area.

These findings have important theoretical implications for psychological contract theory, demonstrating its applicability and relevance in contemporary work arrangements that challenge traditional employment relationship assumptions. The results also provide valuable practical insights for organizations implementing co-working strategies, highlighting the importance of supporting psychological contract reconstruction processes to maintain employee commitment in these new work environments.

## 5 Conclusion

This study provides comprehensive evidence for the mechanisms through which co-working modes influence employee psychological contract reconstruction and subsequent organizational commitment outcomes. The empirical findings demonstrate that co-working environmental characteristics significantly impact psychological contract reconstruction through multiple pathways, including enhanced work autonomy, expanded social networks, and modified organizational support structures. The research reveals that psychological contract reconstruction serves as a critical mediating mechanism that explains how co-working environments translate into organizational commitment, with differential effects across the three commitment dimensions.

The core findings indicate that psychological contract reconstruction has the strongest influence on affective commitment, moderate effects on normative commitment, and weaker but significant impacts on continuance commitment. This pattern suggests that reconstruction processes primarily operate through emotional and identification-based mechanisms rather than cost-benefit calculations, highlighting the importance of addressing psychological and relational aspects of employee-organization relationships in co-working implementations. The moderating role of work autonomy further demonstrates that individual characteristics interact with environmental factors to shape reconstruction outcomes.

The theoretical contributions of this research advance psychological contract theory in three distinct and novel ways that extend beyond incremental theory application. First, this study is the first empirical investigation to identify and test psychological contract reconstruction as a critical mediating mechanism linking co-working environmental characteristics to organizational commitment, establishing that reconstruction processes explain the complex pathways through which shared workspace arrangements influence employee attitudes and behaviors. Prior research examined psychological contracts in traditional organizational settings or focused on contract violations in changing work contexts, but no previous study has systematically investigated reconstruction mechanisms in environments characterized by fluid organizational boundaries and multiple organizational affiliations (Guest, 2004; Fayard and Weeks, 2025). Second, this research reveals that psychological contract reconstruction operates through differentiated pathways for distinct commitment dimensions, with strong effects on affective commitment (β = 0.563), moderate effects on normative commitment (β = 0.431), and weaker effects on continuance commitment (β = 0.189). This pattern contradicts assumptions that psychological contracts uniformly influence all commitment forms and instead demonstrates that reconstruction processes primarily operate through emotional and moral mechanisms rather than calculative pathways, advancing theoretical understanding of commitment formation in contemporary work arrangements. Third, this study identifies a notably weaker-than-expected moderating effect of work autonomy (β = 0.156, *p* < 0.01) on the relationship between co-working environments and psychological contract reconstruction. This finding challenges prevailing assumptions that autonomy universally facilitates adaptation to new work arrangements and suggests that other factors—potentially including psychological maturity, resilience, or age-related adaptability—may play more substantial roles in determining successful psychological contract reconstruction in co-working contexts. This unexpected finding opens important avenues for future research examining individual differences that enable or constrain adaptation to shared workspace environments, representing a significant theoretical contribution that advances understanding of boundary conditions for psychological contract reconstruction processes. The research also contributes to organizational commitment literature by identifying psychological contract reconstruction as a novel antecedent that operates through unique mechanisms in shared workspace environments.

From a practical perspective, organizational managers should prioritize supporting psychological contract reconstruction processes when implementing co-working strategies. Key recommendations include developing clear communication protocols to help employees understand changing expectations, providing adequate organizational support that compensates for reduced physical presence, and designing co-working arrangements that enhance rather than undermine employee autonomy. Organizations should also recognize that reconstruction is an ongoing process requiring sustained attention rather than one-time interventions.

The management implications suggest that successful co-working implementations require proactive psychological contract management, including regular assessment of employee expectations, transparent communication about organizational commitments, and adaptive support systems that respond to the unique challenges of shared workspace environments. Organizations should also invest in building social networks and community connections within co-working spaces to facilitate positive reconstruction outcomes.

Several limitations constrain the generalizability and interpretation of these findings. The cross-sectional design prevents causal inferences about the directionality of relationships between co-working environments, psychological contract reconstruction, and organizational commitment. A particularly important limitation concerns the co-working experience duration of participants. The majority of participants (41.4%) had worked in co-working environments for only 6–12 months, with an additional 34.2% having 1–2 years of experience. This relatively short tenure in co-working arrangements limits our ability to assess long-term psychological contract reconstruction effects. Psychological contract reconstruction is theoretically conceptualized as a dynamic, iterative process that evolves as employees gain experience with new work arrangements and receive ongoing feedback about organizational obligations (Tomprou et al., 2015). Employees in the early stages of co-working experience (6–12 months) may still be actively negotiating their reconstructed contracts and have not yet reached stable equilibrium states. The stronger effects observed on affective commitment compared to continuance commitment might partly reflect the temporal dynamics of reconstruction, where emotional responses emerge more quickly than calculative assessments of switching costs. Future longitudinal research tracking employees over 2–3 years in co-working environments is essential to understand how psychological contract reconstruction processes evolve, stabilize, or transform over time, and whether the commitment patterns observed in this study persist or change as co-working experience accumulates. Additionally, the limited representation of older workers (15.6% over age 41) restricts understanding of age-related differences in reconstruction processes, and the focus on specific metropolitan areas may limit external validity across different cultural and economic contexts.

Future research should employ longitudinal designs to examine the temporal dynamics of psychological contract reconstruction and investigate how reconstruction processes evolve as employees gain experience with co-working arrangements (Bal et al., 2008; Wang et al., 2021). Specific research directions include: (1) conducting longitudinal studies tracking employees over 2–3 years to understand how psychological contracts stabilize or transform in co-working contexts, (2) investigating individual differences including age, psychological maturity, resilience, and personality traits that facilitate or hinder successful reconstruction, (3) examining how organizations can proactively support reconstruction through communication strategies, organizational support mechanisms, and autonomy-enhancing practices, (4) exploring cultural variations in reconstruction processes across different national and organizational cultures, (5) investigating the role of technology and virtual communication tools in facilitating psychological contract development when physical organizational presence is reduced, (6) examining how multiple organizational affiliations characteristic of co-working environments affect contract reconstruction and commitment development, and (7) conducting comparative studies examining psychological contract reconstruction across different flexible work arrangements including fully remote work, hybrid models, and co-working spaces to identify unique features and common mechanisms. This multifaceted research agenda will advance theoretical understanding while providing practical guidance for organizations navigating the continuing evolution toward flexible, shared, and hybrid work arrangements that characterize post-pandemic workplace landscapes.

Future research directions include examining cultural variations in reconstruction processes, investigating the role of technology in facilitating virtual psychological contract development, and exploring how multiple organizational affiliations affect contract reconstruction in complex co-working arrangements. This research contributes valuable insights for promoting the healthy development of co-working modes by demonstrating the critical importance of psychological factors in determining the success of these innovative work arrangements and providing evidence-based guidance for their effective implementation.

## Data Availability

The original contributions presented in the study are included in the article/Supplementary material, further inquiries can be directed to the corresponding author.
