# Supplementary material for: Psychological contract reconstruction in co-working modes and its impact on organizational commitment: an empirical study
Source: Front Psychol. 2026 Jan 21;16:1677068. doi: 10.3389/fpsyg.2025.1677068 (PMC12867845; doi:10.3389/fpsyg.2025.1677068)
Supplement: Supplementary file 1 [file Data_Sheet_1.docx]

**Appendix A: Mathematical Specifications**

**A.1 Reliability and Validity Measures**

**A.1.1 Cronbach's Alpha Coefficient**

Internal consistency reliability was assessed using Cronbach's alpha coefficient, calculated as:

**α = (k/(k-1)) × (1 - Σσ²ᵢ/σ²ₜ)**

Where:

- α = Cronbach's alpha coefficient
- k = number of items in the scale
- σ²ᵢ = variance of item i
- σ²ₜ = total variance of the scale
- Σ = summation across all items

Acceptable threshold: α ≥ 0.70 for research purposes

**A.1.2 Composite Reliability (CR)**

Composite reliability for each latent construct was computed as:

**CR = (Σλᵢ)² / [(Σλᵢ)² + Σ(1-λ²ᵢ)]**

Where:

- CR = composite reliability
- λᵢ = standardized factor loading of item i
- Σλᵢ = sum of standardized factor loadings
- Σ(1-λ²ᵢ) = sum of error variances

Acceptable threshold: CR ≥ 0.70

**A.1.3 Average Variance Extracted (AVE)**

Average variance extracted for convergent validity was calculated as:

**AVE = Σλ²ᵢ / n**

Where:

- AVE = average variance extracted
- λ²ᵢ = squared standardized factor loading of item i
- n = number of items in the construct
- Σ = summation across all items

Acceptable threshold: AVE ≥ 0.50

**A.1.4 Discriminant Validity (Fornell-Larcker Criterion)**

Discriminant validity was assessed by comparing the square root of AVE with inter-construct correlations:

**√AVEᵢ > rᵢⱼ**

Where:

- √AVEᵢ = square root of average variance extracted for construct i
- rᵢⱼ = correlation coefficient between construct i and construct j

Criterion met when: √AVE for each construct exceeds all its correlations with other constructs

**A.2 Psychological Contract Reconstruction Measurement Model**

**A.2.1 Psychological Contract Reconstruction (PCR) Formula**

The measurement of psychological contract reconstruction conceptualizes four sequential components:

**PCR = α(ED) + β(SM) + γ(EA) + δ(CR) + ε**

Where:

- PCR = Psychological Contract Reconstruction (latent variable)
- ED = Environmental Disruption component
- SM = Sense-Making processes component
- EA = Expectation Adjustment component
- CR = Contract Reformulation component
- α, β, γ, δ = weighting coefficients (path coefficients)
- ε = measurement error term

The weighting coefficients are determined through confirmatory factor analysis, representing the relative contribution of each component to the overall reconstruction process.

**A.3 Organizational Commitment Composite Measurement**

**A.3.1 Overall Organizational Commitment Formula**

Following Allen and Meyer's (1990) three-component model, overall organizational commitment combines three dimensions:

**OC = w₁(AC) + w₂(CC) + w₃(NC)**

Where:

- OC = Overall Organizational Commitment
- AC = Affective Commitment
- CC = Continuance Commitment
- NC = Normative Commitment
- w₁, w₂, w₃ = dimension weights

Dimension weights are determined through confirmatory factor analysis and standardized to sum to 1.0, reflecting the relative importance of each commitment dimension in the overall construct.

**A.4 Mediation Effect Testing**

**A.4.1 Indirect Effect Formula (Hayes' Method)**

The mediation effect of psychological contract reconstruction was tested using Hayes' (2017) bias-corrected bootstrap method:

**Indirect Effect (IE) = a × b**

Where:

- IE = indirect effect (mediation effect)
- a = path coefficient from independent variable (co-working environment) to mediator (psychological contract reconstruction)
- b = path coefficient from mediator (psychological contract reconstruction) to dependent variable (organizational commitment)

**A.4.2 Standard Error of Mediation Effect**

The standard error of the mediation effect was calculated as:

**SE(ab) = √(a²s²ᵦ + b²s²ₐ + s²ₐs²ᵦ)**

Where:

- SE(ab) = standard error of the mediation effect
- a = path coefficient from IV to mediator
- b = path coefficient from mediator to DV
- s²ₐ = variance (squared standard error) of path a
- s²ᵦ = variance (squared standard error) of path b

**A.4.3 Significance Testing of Mediation Effect**

The z-statistic for testing significance of the mediation effect:

**z = (a × b) / SE(ab)**

**z = (a × b) / √(a²s²ᵦ + b²s²ₐ + s²ₐs²ᵦ)**

Where all terms are as defined above.

Significance determined when 95% bias-corrected bootstrap confidence interval excludes zero, based on 5,000 bootstrap resamples.

**A.4.4 Bootstrap Confidence Interval**

95% bias-corrected bootstrap confidence interval for indirect effect:

**CI₉₅ = [IE - 1.96 × SE(ab), IE + 1.96 × SE(ab)]**

Mediation is supported when the confidence interval does not include zero.

**A.5 Structural Equation Modeling (SEM) Specifications**

**A.5.1 Basic SEM Equation System**

The structural equation model framework follows the general form:

**η = Βη + Γξ + ζ**

Where:

- η = vector of endogenous latent variables (psychological contract reconstruction, affective commitment, continuance commitment, normative commitment)
- ξ = vector of exogenous latent variables (co-working environmental characteristics, work autonomy)
- Β = matrix of structural coefficients among endogenous variables (path coefficients between mediator and outcomes)
- Γ = matrix of structural coefficients from exogenous to endogenous variables (path coefficients from predictors to mediator and outcomes)
- ζ = vector of structural equation errors (residuals)

**A.5.2 Measurement Model Equations**

The relationship between latent variables and observed indicators:

**X = Λₓξ + δ**

**Y = Λᵧη + ε**

Where:

- X = vector of observed indicators for exogenous variables
- Y = vector of observed indicators for endogenous variables
- Λₓ = matrix of factor loadings for exogenous indicators
- Λᵧ = matrix of factor loadings for endogenous indicators
- δ = vector of measurement errors for X
- ε = vector of measurement errors for Y

**A.5.3 Moderation Effect Testing**

The moderating effect of work autonomy was tested by including an interaction term:

**PCR = β₀ + β₁(CWE) + β₂(WA) + β₃(CWE × WA) + ε**

Where:

- PCR = Psychological Contract Reconstruction
- CWE = Co-working Environmental characteristics
- WA = Work Autonomy (moderator)
- CWE × WA = interaction term (product of standardized variables)
- β₀ = intercept
- β₁ = main effect of co-working environment
- β₂ = main effect of work autonomy
- β₃ = moderation effect (interaction coefficient)
- ε = error term

Significant moderation is indicated when β₃ is statistically significant (p < 0.05).

**A.6 Model Fit Indices**

**A.6.1 Chi-Square Statistic**

**χ² = Σ[(Observed - Expected)² / Expected]**

Where:

- χ² = chi-square test statistic
- Observed = observed covariance matrix
- Expected = model-implied covariance matrix

Relative chi-square: **χ²/df < 3.0** indicates acceptable fit

**A.6.2 Comparative Fit Index (CFI)**

**CFI = 1 - [(χ²ₜ - dfₜ) / (χ²ᵦ - dfᵦ)]**

Where:

- χ²ₜ = chi-square for target model
- χ²ᵦ = chi-square for baseline model
- df = degrees of freedom

Acceptable threshold: CFI ≥ 0.90; good fit: CFI ≥ 0.95

**A.6.3 Tucker-Lewis Index (TLI)**

**TLI = [(χ²ᵦ/dfᵦ) - (χ²ₜ/dfₜ)] / [(χ²ᵦ/dfᵦ) - 1]**

Acceptable threshold: TLI ≥ 0.90; good fit: TLI ≥ 0.95

**A.6.4 Root Mean Square Error of Approximation (RMSEA)**

**RMSEA = √[(χ² - df) / (df × N)]**

Where:

- N = sample size
- χ² = chi-square statistic
- df = degrees of freedom

Acceptable threshold: RMSEA ≤ 0.08; good fit: RMSEA ≤ 0.06

90% Confidence Interval should be reported

**A.6.5 Standardized Root Mean Square Residual (SRMR)**

**SRMR = √[Σ(rᵢⱼ - r̂ᵢⱼ)² / k]**

Where:

- rᵢⱼ = observed correlation between variables i and j
- r̂ᵢⱼ = model-implied correlation between variables i and j
- k = number of unique correlations

Acceptable threshold: SRMR ≤ 0.08; good fit: SRMR ≤ 0.05

**A.7 Effect Size Interpretation**

**A.7.1 Cohen's Guidelines for Path Coefficients**

Effect size interpretation for standardized path coefficients (β):

- **Small effect:** |β| = 0.10 to 0.30 (R² ≈ 1% to 9%)
- **Medium effect:** |β| = 0.30 to 0.50 (R² ≈ 9% to 25%)
- **Large effect:** |β| > 0.50 (R² > 25%)

**A.7.2 Variance Explained Calculation**

Approximate variance explained by a path coefficient:

**R² ≈ β²**

Where:

- R² = proportion of variance explained
- β = standardized path coefficient

More precisely, for multiple predictors:

**R² = 1 - (σ²ε / σ²y)**

Where:

- σ²ε = residual variance
- σ²y = total variance in dependent variable

**A.8 Statistical Power Analysis**

**A.8.1 Minimum Sample Size Calculation**

Minimum required sample size for SEM was determined using:

**N = 384** for medium effect size (f² = 0.15), α = 0.05, power = 0.80

Based on the formula:

**N ≥ (Zα + Zβ)² × (1/f²) + 3**

Where:

- Zα = z-score for significance level (1.96 for α = 0.05)
- Zβ = z-score for statistical power (0.84 for power = 0.80)
- f² = effect size (Cohen's f²)

**A.9 Bootstrap Resampling Specifications**

**A.9.1 Bias-Corrected Bootstrap Method**

Bootstrap specifications for mediation analysis:

- Number of bootstrap samples: **5,000**
- Confidence level: **95%**
- Method: **Bias-corrected percentile method**
- Random seed: Set for reproducibility

The bias-corrected confidence interval adjusts for potential bias in the bootstrap distribution:

**CI_BC = [θ̂_α₁, θ̂_α₂]**

Where:

- α₁ and α₂ are adjusted percentile values
- Adjustment based on the proportion of bootstrap estimates less than the original estimate

**References for Appendix**

Anderson, J. C., & Gerbing, D. W. (1988). Structural equation modeling in practice: A review and recommended two-step approach. Psychological Bulletin, 103(3), 411-423.

Cohen, J. (1988). Statistical power analysis for the behavioral sciences (2nd ed.). Hillsdale, NJ: Lawrence Erlbaum Associates.

Fornell, C., & Larcker, D. F. (1981). Evaluating structural equation models with unobservable variables and measurement error. Journal of Marketing Research, 18(1), 39-50.

Hayes, A. F. (2017). Introduction to mediation, moderation, and conditional process analysis: A regression-based approach (2nd ed.). New York: Guilford Press.

Hu, L. T., & Bentler, P. M. (1999). Cutoff criteria for fit indexes in covariance structure analysis: Conventional criteria versus new alternatives. Structural Equation Modeling, 6(1), 1-55.

Kline, R. B. (2016). Principles and practice of structural equation modeling (4th ed.). New York: Guilford Press.

**End of Appendix A**
